# Oxidation resistance 1 is a novel senolytic target

**DOI:** 10.1111/acel.12780

**Published:** 2018-05-15

**Authors:** Xin Zhang, Suping Zhang, Xingui Liu, Yingying Wang, Jianhui Chang, Xuan Zhang, Samuel G. Mackintosh, Alan J. Tackett, Yonghan He, Dongwen Lv, Remi‐Martin Laberge, Judith Campisi, Jianrong Wang, Guangrong Zheng, Daohong Zhou

**Affiliations:** ^1^ Department of Pharmaceutical Sciences College of Pharmacy University of Arkansas for Medical Sciences Little Rock Arkansas; ^2^ Hematology Center of Cyrus Tang Medical Institute Collaborative Innovation Center of Hematology Soochow University School of Medicine Suzhou China; ^3^ Department of Biochemistry and Molecular Biology College of Medicine University of Arkansas for Medical Sciences Little Rock Arkansas; ^4^ Unity Biotechnology Brisbane California; ^5^ The Buck Institute for Research on Aging Novato California; ^6^ Lawrence Berkeley National Laboratories Berkeley California; ^7^ Department of Medicinal Chemistry College of Pharmacy University of Florida Gainesville Florida; ^8^ Department of Pharmcodynamics College of Pharmacy University of Florida Gainesville Florida

**Keywords:** cellular senescence, OXR1, piperlongumine, reactive oxygen species

## Abstract

The selective depletion of senescent cells (SCs) by small molecules, termed senolytic agents, is a promising therapeutic approach for treating age‐related diseases and chemotherapy‐ and radiotherapy‐induced side effects. Piperlongumine (PL) was recently identified as a novel senolytic agent. However, its mechanism of action and molecular targets in SCs was unknown and thus was investigated. Specifically, we used a PL‐based chemical probe to pull‐down PL‐binding proteins from live cells and then mass spectrometry‐based proteomic analysis to identify potential molecular targets of PL in SCs. One prominent target was oxidation resistance 1 (OXR1), an important antioxidant protein that regulates the expression of a variety of antioxidant enzymes. We found that OXR1 was upregulated in senescent human WI38 fibroblasts. PL bound to OXR1 directly and induced its degradation through the ubiquitin‐proteasome system in an SC‐specific manner. The knockdown of *OXR1* expression by RNA interference significantly increased the production of reactive oxygen species in SCs in conjunction with the downregulation of antioxidant enzymes such as heme oxygenase 1, glutathione peroxidase 2, and catalase, but these effects were much less significant when *OXR1* was knocked down in non‐SCs. More importantly, knocking down *OXR1* selectively induced apoptosis in SCs and sensitized the cells to oxidative stress caused by hydrogen peroxide. These findings provide new insights into the mechanism by which SCs are highly resistant to oxidative stress and suggest that OXR1 is a novel senolytic target that can be further exploited for the development of new senolytic agents.

## 1 INTRODUCTION

Cellular senescence occurs when irreversible cell cycle arrest is triggered by telomere shortening or exposure to stress (Campisi, 2013). The induction of cellular senescence has many beneficial effects, including preventing tumorigenesis, promoting wound healing and tissue remodeling, and contributing to embryonic development (Muñoz‐Espín & Serrano, 2014). However, senescent cells (SCs) accumulate if they cannot be removed rapidly by the immune system due to immune dysfunction and/or a sustained, overwhelming increase in SC production. This occurs during aging or under certain pathological conditions (Childs et al., 2017; Muñoz‐Espín & Serrano, 2014). Under these circumstances, SCs can be detrimental and play a causal role in aging, age‐related diseases, and chemotherapy‐ and radiotherapy‐induced side effects, in part through the expression of the senescence‐associated secretory phenotype (Childs et al., 2017; Muñoz‐Espín & Serrano, 2014). This hypothesis is supported by recent studies demonstrating that the genetic clearance of SCs prolongs the lifespan of mice and delays the onset of several age‐related diseases and disorders in both progeroid and naturally aged mice (Baker et al., 2011, 2016). Therefore, the pharmacological clearance of SCs with a small molecule, a senolytic agent that can selectively kill SCs, is potentially a novel anti‐aging strategy and a new treatment for chemotherapy‐ and radiotherapy‐induced side effects (Baar et al., 2017; Chang et al., 2016; Childs et al., 2016; Demaria et al., 2017; Jeon et al., 2017; Ogrodnik et al., 2017; Pan et al., 2017; Schafer et al., 2017; Yosef et al., 2016; Zhu et al., 2015).

However, a major challenge facing the discovery and development of effective senolytic agents is to identify and validate more senolytic targets. Since the first senolytic was published (Zhu et al., 2015), twelve molecular targets have been identified (Childs et al., 2017), including the prosurvival Bcl‐2 family proteins (Chang et al., 2016; Yosef et al., 2016; Zhu et al., 2016) and forkhead Box O4 (FOXO4) (Baar et al., 2017). These findings led to the discovery of a few senolytic agents, including ABT‐263 and ABT‐737, two Bcl‐2/xl/w inhibitors, and FOXO4‐DRI, a peptide molecule that interferes with the interaction of FOXO4 and p53. Unfortunately, the clinical application of these senolytic agents for age‐related diseases and cytotoxic cancer therapy‐induced side effects may be limited because of the toxicity of Bcl‐2/xl/w inhibitors and the difficulty of developing effective high molecular‐weight peptide therapeutics. Therefore, identification of new SC molecular targets is in urgent need for the development of novel small molecule senolytic agents.

Piperlongumine (PL) is one of a few natural products identified to have the ability to selectively kill SCs (Wang et al., 2016; Zhu et al., 2015, 2017). Compared to other known senolytic agents, PL has the advantage of low toxicity, an excellent PK/PD profile, and oral bioavailability (Raj et al., 2011). However, PL has a relatively higher EC_50_ value against SCs compared with ABT‐263 (Wang et al., 2016). Moreover, its molecular target(s) and mechanism of action are unknown. To facilitate the development of PL and its analogues as senolytic drug candidates, it is critical to identify PL molecular targets, which can form a molecular basis for the rational design of new PL analogues.

Herein, we report the identification and validation of oxidation resistance 1 (OXR1) as a molecular target of PL in SCs. OXR1 is a cellular oxidative stress sensor that regulates the expression of a variety of antioxidant enzymes and modulates the cell cycle and apoptosis (Oliver et al., 2011; Yang et al., 2015). We found that OXR1 was upregulated in SCs induced by ionizing radiation (IR) or extensive replication. PL bound to OXR1 directly and induced its degradation through the ubiquitin‐proteasome system in an SC‐specific manner. Knocking down *OXR1* selectively induced apoptosis in SCs and sensitized the cells to oxidative stress caused by hydrogen peroxide (H_2_O_2_). These findings suggest that OXR1 is a potential senolytic target that can be exploited for the development of selective senolytic agents with improved potency and selectivity. In addition, these findings also provide new insight into the mechanism by which SCs are highly resistant to oxidative stress.

## 2 RESULTS

### 2.1 Design and synthesis of PL probes

**Figure 1:**
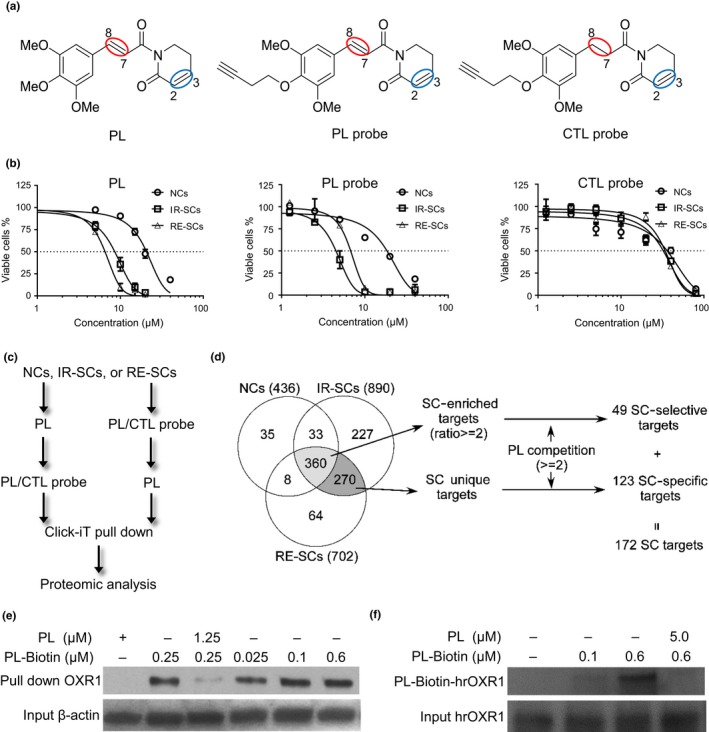
Identification and validation of OXR1 as a target of PL. (a) The chemical structures of piperlongumine (PL), alkyne‐labeled piperlongumine (PL probe), and alkyne‐labeled negative control probe (CTL probe). The C2–C3 bonds are circled in blue, and the C7–C8 bonds are circled in red. (b) Viable cell counts of nonsenescent WI‐38 cells (NCs), IR‐induced senescent WI‐38 cells (IR‐SC), and replication‐exhausted senescent WI‐38 cells (RE‐SC) 72 hr after treatment with PL, the PL probe, or the CTL probe. Data are presented as the mean ± SE of percent cell viability from two independent experiments. (c) Schematic of the competitive pull‐down procedure by “Click” Chemistry. (d) Numbers of PL‐binding proteins identified in NCs, IR‐SCs, and RE‐SCs. The list of the proteins is presented in Supporting Information Tables S1 and S2. (e) IR‐SC lysate was incubated with biotin‐labeled PL in the absence or presence of a fivefold excess of unlabeled PL followed by precipitation with streptavidin‐conjugated agarose beads and immunoblotting of the proteins released from the beads with anti‐OXR1 antibodies. An immunoblot of β‐actin in IR‐SC lysates was included as an input control for the pull down. (f) Human recombinant OXR1 protein was incubated with biotin‐labeled PL in the absence or presence of a fivefold excess of unlabeled PL followed by immunoblotting with antibodies against biotin to detect OXR1 bound to the biotin‐labeled PL probe. An immunoblot of OXR1 from each reaction was included as an input control

The structure of PL features two Michael acceptors (electrophiles), the C2–C3 and C7–C8 olefins (Figure 1a) (Adams et al., 2012). Our previous studies showed that both Michael acceptors were important for the senolytic activity of PL (Wang et al., 2016), indicating that PL may act as an irreversible inhibitor through the conjugated addition of the nucleophiles (e.g., cysteine residue) on its target protein(s) to the Michael acceptors. Taking advantage of the covalent interaction between PL and its target protein(s), we designed PL probes that can be “tagged” for protein purification and identification in live cells (Figure 1a). Our structure–activity relationship studies revealed that modifications on the trimethoxyphenyl group of PL were tolerated, and an affinity tag could be attached to the para‐position of the phenyl ring. We thus synthesized PL probes that contain a terminal alkyne. These probes can be used to enrich target proteins by reacting with the corresponding immobilized azide through a bio‐orthogonal copper‐catalyzed azide–alkyne cycloaddition (Click Chemistry) (Supporting Information Figure S1) (Kolb & Sharpless, 2003), which allows us to perform mass spectrometry (MS)‐based proteomic analysis to identify the target proteins. Through a cell‐based senolytic activity assay (Figure 1b), we selected a PL probe (Figure 1a) with the same senolytic activity as PL to be used in our target‐protein identification study. As a negative control, we designed a structurally related compound in which the C7–C8 double bond of the PL probe is saturated (CTL probe, Figure 1a); we modified this bond because previous studies suggested that the Michael acceptor at the PL C7–C8 olefin covalently binds target proteins, while the C2–C3 olefin can facilitate target‐protein binding after reacting with glutathione (Adams et al., 2012; Raj et al., 2011). The CTL probe was used to subtract nonspecific proteins pulled down by our PL probe. As expected, the CTL probe was ~9‐fold less potent than the PL probe and has no selective toxicity against SCs because the senolytic activity of PL depends on the presence of both Michael acceptors (Figure 1b) (Wang et al., 2016).

### 2.2 Identification and validation of OXR1 as a target of PL

To identify the senolytic targets of PL, we conducted pull‐down experiments by incubating the PL probe or CTL probe with normal or nonsenescent WI38 cells (NCs), IR‐induced senescent WI38 cells (IR‐SCs), or replicative senescent WI38 cells (RE‐SCs) as described in method section. To further exclude proteins that bound nonspecifically to PL probe, we also carried out competitive‐binding experiments in which IR‐SCs were incubated with an excess of PL for 2 hr then with the PL probe for 3 hr or vice versa (Figure 1c). After excluding proteins that were bound by the CTL probe, we identified 436 proteins in NCs that bound the PL probe, 890 proteins in IR‐SCs, and 702 proteins in RE‐SCs (Figure 1d). Among these, 270 proteins were unique to both IR‐SCs and RE‐SCs, and 360 proteins were common to all three cell types. From the 360 common proteins, we identified 49 SC‐selective targets that had an enrichment ratio of ≥2 (i.e., the LFQ intensity of proteins in IR‐SCs and RE‐SCs was ≥2‐fold higher than those in NCs) and a PL competition ratio of ≥2 (i.e., the LFQ intensity of proteins on cells treated with the PL probe followed by PL was ≥2‐fold higher than those in the cells treated with PL followed by the PL probe). Similarly, using a PL competition ratio of ≥2 as a cutoff, 123 SC‐unique proteins were retained on the final list as SC‐specific targets (Figure 1d; for the protein list, see Supporting Information Tables S1 and S2).

Overall, we identified 172 potential senolytic targets (123 SC‐specific targets plus 49 SC‐selective targets) of PL. Gene ontology (GO) analysis revealed that these PL targets were mainly involved in the regulation of protein transport and localization, autophagy, TOR signaling, protein phosphorylation, and ubiquitination (Supporting Information Table S3). KEGG pathway analysis revealed that the PL‐target proteins were mainly enriched in endocytosis, phosphatidylinositol signaling system, inositol phosphate metabolism, mTOR signaling pathway, and insulin signaling pathway (Supporting Information Table S4).

We found that OXR1 was on the top of the list of the PL‐binding proteins in SCs (Supporting Information Table S2). OXR1 is crucial for protecting cells against oxidative stress by regulating the expression of several enzymes that detoxify ROS (Jaramillo‐Gutierrez, Molina‐Cruz, Kumar & Barillas‐Mury, 2010; Oliver et al., 2011; Yang et al., 2015), and it may be particularly important as a senolytic target of PL because SCs are known to produce high levels of ROS but remain resistant to oxidative stress (Supporting Information Figure S2) (Chandrasekaran, Idelchik & Melendez, 2017; Davalli, Mitic, Caporali, Lauriola & D'Arca, 2016; Lee et al., 1999; Lu & Finkel, 2008). In addition, LMD‐3, homolog of OXR1 in *Caenorhabditis elegans*, has been shown to protect against oxidative stress and accelerated aging in the worm (Sanada et al., 2014). Therefore, we validated whether PL could bind OXR1 directly using a biotin‐labeled PL probe (Raj et al., 2011). This PL probe pulled down OXR1 from SC lysates and bound directly to recombinant human OXR1 (rhOXR1) in a dose‐dependent manner, which was prevented with an excess of PL (Figure 1e, f). Taken together, these results demonstrate that PL binds OXR1 directly, suggesting that OXR1 may be a senolytic target of PL.

### 2.3 PL selectively reduces the level of OXR1 in IR‐SCs by inducing its degradation

**Figure 2:**
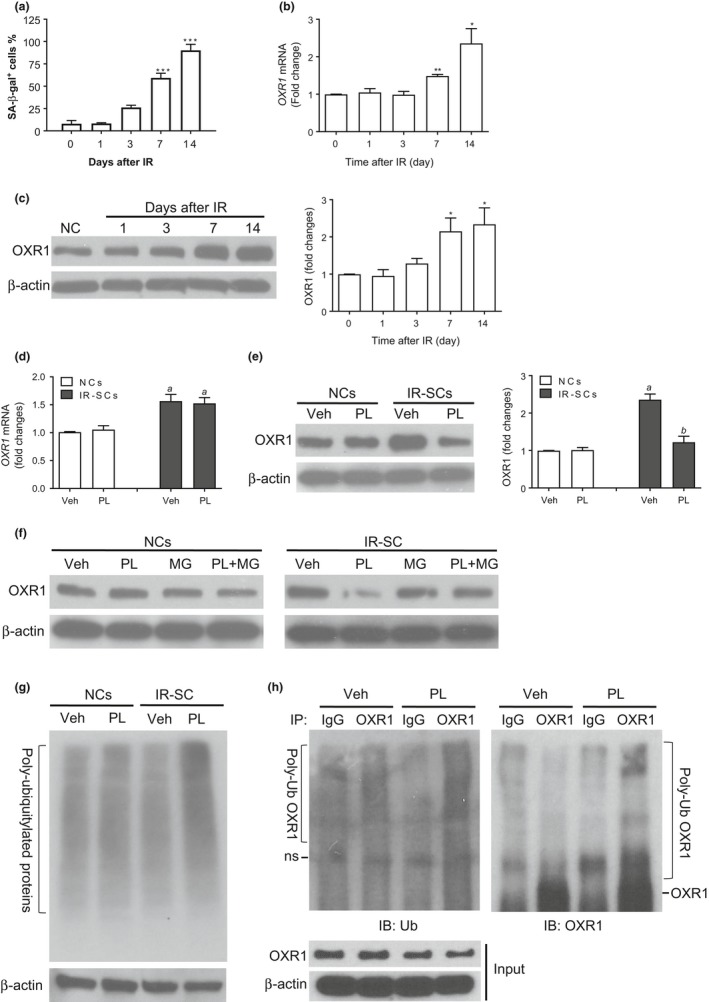
PL selectively decreases the level of OXR1 in SCs, in part by inducing OXR1 proteasomal degradation. (a) Percentage of SA‐β‐gal–positive cells at different days after IR. (b) The levels of *OXR1* mRNA in WI‐38 cells at different days after IR are presented as a fold change from un‐irradiated control cells. (c) The levels of OXR1 protein in WI‐38 cells at different days after IR. Left panel, representative immunoblot of OXR1 and β‐actin; right panel, fold changes in OXR1 expression in irradiated cells relative to un‐irradiated cells after normalization to β‐actin. (d) The levels of *OXR1* mRNA in NCs and IR‐SCs after incubation with vehicle (Veh) or PL (5 μM) for 6 hr. (e) The levels of OXR1 protein in NCs and IR‐SCs after incubation with vehicle (Veh) or PL (5 μM) for 6 hr. Left panel, representative immunoblot of OXR1 and β‐actin; right panel, fold changes in OXR1 expression in cells relative to vehicle‐treated NCs after normalization to β‐actin. (f) The levels of OXR1 were determined in NCs and IR‐SCs after incubation with vehicle (Veh) or PL (5 μM) for 6 hr in the presence or absence of 5 μM MG‐132 (MG). (g) The levels of poly‐ubiquitylated (Poly‐Ub) proteins in NCs and IR‐SCs after incubation with vehicle (Veh) or PL (5 μM) for 6 hr; β‐actin was included as a loading control. (h) The levels of poly‐ubiquitylated (Poly‐Ub) OXR1 in IR‐SCs after incubation with vehicle (Veh) or PL (5 μM) for 6 hr; the cell lysates were incubated with OXR1 or IgG control and resin for overnight. After extensive washing, ubiquitinated OXR1 was eluted by SDS sample buffer and immunoblotted with ubiquitin and OXR1 antibodies. The input cell lysates were also immunoblotted with antibodies to OXR1 or β‐actin as indicated. ns indicates the nonspecific band. Data in bar graphs are the mean ± SE of two to three independent experiments. *p <* *0.05, **p <* *0.01, and ***p <* *0.001 vs. un‐irradiated cells by one‐way ANOVA. ^a^ *p *<* *0.05 vs. NCs treated with Veh; ^b^ *p *<* *0.05 vs. IR‐SCs treated with Veh by two‐way ANOVA

To further confirm that OXR1 is a senolytic target of PL, we examined the effect of PL on *OXR1* expression in NCs and IR‐SCs. We found that IR induced a time‐dependent increase in *OXR1* mRNA and OXR1 protein in WI‐38 cells, which correlated closely with the time‐dependent induction of senescence as determined by senescence‐associated β‐galactosidase staining (Figure 2a–c). PL treatment had no significant effect on the levels of *OXR1* mRNA and protein in NCs (Figure 2d, e). In contrast, PL substantially reduced the levels of OXR1 protein but had no effect on *OXR1* mRNA expression in IR‐SCs, suggesting that PL regulates OXR1 in IR‐SCs post‐transcriptionally, possibly by inducing its degradation. Indeed, the PL‐induced downregulation of OXR1 was attenuated by suppressing the activity of the proteasome with MG‐132 (Figure 2f). Moreover, we found that PL selectively increased the levels of poly‐ubiquitylated proteins in IR‐SCs, but not in NCs (Figure 2g); and IR‐SCs treated with PL had higher levels of both mono‐ubiquitinated OXR1 and poly‐ubiquitylated OXR1 than those treated with vehicle (Figure 2h). Collectively, these results suggest that PL can selectively reduce the levels of OXR1 in SCs, in part by inducing the proteasomal degradation of OXR1.

### 2.4 More intracellular PL in IR‐SCs than NCs

**Figure 3:**
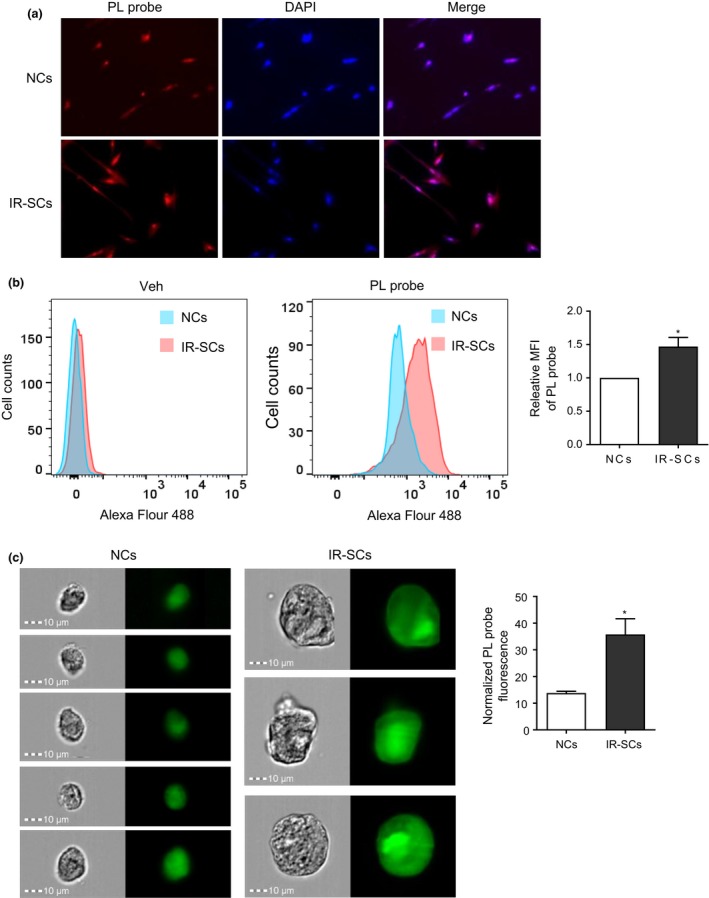
IR‐SCs have higher levels of PL than NCs. NCs and IR‐SCs were incubated with vehicle (Veh) or alkyne‐labeled PL probe (5 μM) for 5 hr, and probe uptake was detected with Alexa Fluor azide 488. (a) PL probe uptake was visualized by microscopy, (b) measured by flow cytometry, and (c) quantified by ImageStream flow cytometry. (a) DAPI was used for nuclear staining. (b) Left and middle panels, representative histograms of flow cytometric analysis; right panel, relative MFI of Alexa Fluor 488‐PL staining, normalized by cell size. (c) Left and middle panels, representative brightfield and Alexa Fluor 488 fluorescent images of NCs and IR‐SCs after incubation with the PL probe; right panel, normalized relative Alexa Fluor 488 fluorescence intensity in NCs and IR‐SCs according to cell size. Data in the bar graphs are the mean ± SE of two to three independent experiments. *p <* *0.05 vs. NCs by unpaired *t* test

To understand the mechanism by which PL selectively induced the degradation of OXR1 in IR‐SCs, we measured the levels of PL in NCs and IR‐SCs by taking advantage of our alkyne‐bearing PL probe. Specifically, NCs and IR‐SCs were incubated with the PL probe, and its levels were determined by a Click Chemistry reaction with Alexa Fluor 488 azide after the free probe was removed. With fluorescence microscopy, we found that IR‐SCs appeared to have a higher level of the PL probe than NCs (Figure 3a). To quantify the difference in the intracellular levels of the PL probe between NCs and IR‐SCs, we measured the mean fluorescence intensity (MFI) of the Alexa Fluor 488‐PL probe with flow cytometry after the MFI was normalized by cell size according to the forward scatter (FSC) measurement because SCs are bigger than NCs (Figure 3b). We found that IR‐SCs had a significantly higher level of intracellular PL probe than NCs. This finding was also confirmed by analysis with an ImageStream flow cytometer after considering the difference in cell size measured by the average area of cells between NCs and IR‐SCs (Figure 3c). These results suggest that IR‐SCs may uptake and retain more PL or degrade less PL than NCs, which may contribute to the selective induction of OXR1 degradation and cell death in SCs by PL.

### 2.5 Knocking down *OXR1* selectively kills IR‐SCs by downregulating the expression of antioxidant enzymes and sensitizing the cells to oxidative stress

**Figure 4:**
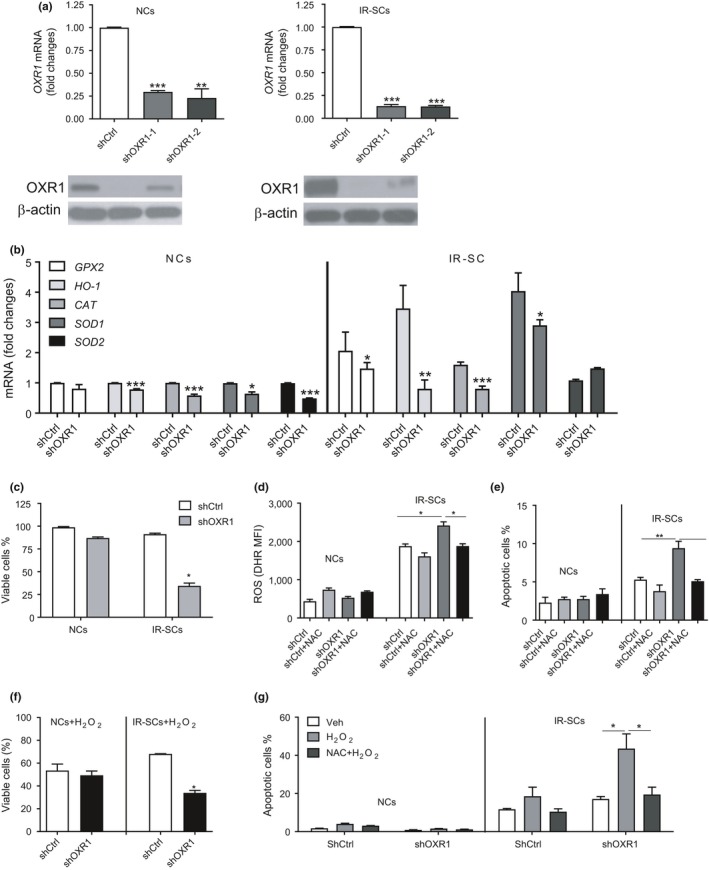
Knocking down *OXR1* selectively kills IR‐SCs by downregulating the expression of antioxidant enzymes to sensitize the cells to oxidative stress. (a) The levels of *OXR1* mRNA (top) and OXR1 protein (bottom) in NCs and IR‐SCs transfected with either scramble control shRNA (shCtrl) or two different shRNA constructs targeting *OXR1* (shOXR1‐1 or shOXR1‐2). (b) Expression of glutathione peroxidase 2 (*GPX2*), heme oxygenase 1 (*HO‐1*), catalase (*CAT*), superoxide dismutase 1 (*SOD1*), and *SOD2* mRNA in NCs and IR‐SCs transfected with shCtrl or shOXR1. Data in (a) and (b) are the mean ± SE of fold changes in mRNA expression compared with shCtrl‐transfected cells from two to three independent experiments. *p <* *0.05, **p <* *0.01, and ***p <* *0.001 vs. shCtrl by unpaired *t* test. (c) Percent viability of NCs and IR‐SCs transfected with shCtrl or shOXR1 presented as the mean ± SE of two independent experiments. *p <* *0.05 vs. IR‐SCs transfected with shCtrl by unpaired *t* test. (d) Levels of intracellular ROS in NCs and IR‐SCs transfected with shCtrl or shOXR1 in the presence or absence of 2 mM *N*‐acetyl‐l‐cysteine (NAC). Data are presented as the mean ± SE of mean fluorescent intensity (MFI) of dihydrorhodamine 123 (DHR) from two independent experiments. *p <* *0.05 vs. IR‐SCs transfected with shOXR1 without NAC by unpaired *t* test. (e) Percent apoptotic cells in NCs and IR‐SCs transfected with shCtrl or shOXR1 in the presence or absence of 2 mM NAC are presented as the mean ± SE of two independent experiments. *p <* *0.05 vs. IR‐SCs transfected with shOXR1 without NAC by unpaired *t* test. (f) Percent viable cells in NCs and IR‐SCs transfected with either shCtrl or shOXR1 after incubation with 100 μM H_2_O_2_ for 3 day. Data are presented as the mean ± SE of percent viable cells compared with their respective controls without H_2_O_2_ treatment from two independent experiments. *p <* *0.05 vs. IR‐SCs transfected with shCtrl by unpaired *t* test. (g) Percent apoptotic cells in NS and IR‐SCs transfected with shCtrl or shOXR1 after incubation with 100 μM H_2_O_2_ in the presence or absence of 2 mM NAC. Data are presented as the mean ± SE of two to three independent experiments. *p <* *0.05 vs. IR‐SCs transfected with shOXR1 with H_2_O_2_ alone by unpaired *t* test

OXR1 is an important antioxidant protein that protects cells from oxidative stress by regulating the expression of enzymes that detoxify ROS, such as glutathione peroxidase 2 (GTX2), heme oxygenase 1 (HO‐1), and catalase (CAT) (Jaramillo‐Gutierrez et al., 2010; Oliver et al., 2011; Yang et al., 2015). SCs are known to produce high levels of ROS, but they are highly resistant to oxidative stress (Chandrasekaran et al., 2017; Davalli et al., 2016; Lee et al., 1999; Lu & Finkel, 2008). This resistance may be due to the increased expression of OXR1 and its downstream targets in SCs; therefore, OXR1 may be a genuine senolytic target. To test this hypothesis, we used two different *OXR1* shRNAs (shOXR1‐1 and shOXR1‐2) to knock down the expression of OXR1 in both NCs and IR‐SCs (Figure 4a). We found that shOXR1‐1 was slightly more effective than shOXR1‐2 at knocking down *OXR1* in both cell types; thus, we used shOXR1‐1 (referred to as shOXR1) for the rest of the study.

As expected, IR‐SCs expressed significantly higher levels of the known downstream targets of OXR1, that is, *GTX2* and *HO‐1* and *CAT*, and other antioxidant enzymes such as superoxide dismutase 1 (*SOD‐1*) and *SOD2* than NCs (Figure 4b). Knocking down *OXR1* slightly reduced the expression of *HO‐1* and *CAT* mRNA but not *GTX2* mRNA, in NCs. In contrast, IR‐SCs showed a greater reduction in *GTX2*,* HO‐1,* and *CAT* mRNA expression after *OXR1* knockdown. Knocking down *OXR1* also moderately reduced the expression of *SOD‐1* and *SOD‐2* mRNA in NCs and *SOD‐1* mRNA in IR‐SCs. Importantly, knocking down *OXR1* significantly reduced the viability of IR‐SCs, but not NCs (Figure 4c). These findings suggest that OXR1 may be a novel senolytic target.

To determine whether *OXR1* knockdown selectively killed IR‐SCs through oxidative stress, we measured ROS production and apoptosis in IR‐SCs and NCs after transfection with control shRNA (shCtrl) or shOXR1 in the presence or absence of the antioxidant *N*‐acetyl‐cysteine (NAC). The IR‐SCs produced significantly higher levels of ROS than NCs, and IR‐SCs produced even more ROS after knockdown of *OXR1* (Figure 4d). This increase in ROS was associated with a significant increase in apoptosis in IR‐SCs after knocking down *OXR1* (Figure 4e). Importantly, the increases in ROS and apoptosis in IR‐SCs induced by shOXR1 were abrogated by NAC. In contrast, knocking down *OXR1* in NCs only slightly increased ROS production, but this had no significant effect on NC apoptosis. These results suggest that knocking down *OXR1* selectively kills IR‐SCs by sensitizing them to oxidative stress and inducing apoptosis. This is supported by our finding that knocking down *OXR1* also sensitized IR‐SCs but not NCs to H_2_O_2_‐induced cell death and apoptosis, and this was abrogated by NAC (Figure 4f, g).

### 2.6 Knockdown of *OXR1* also induces apoptosis in RE‐SCs by downregulating the expression of antioxidant enzymes and increasing ROS production

**Figure 5:**
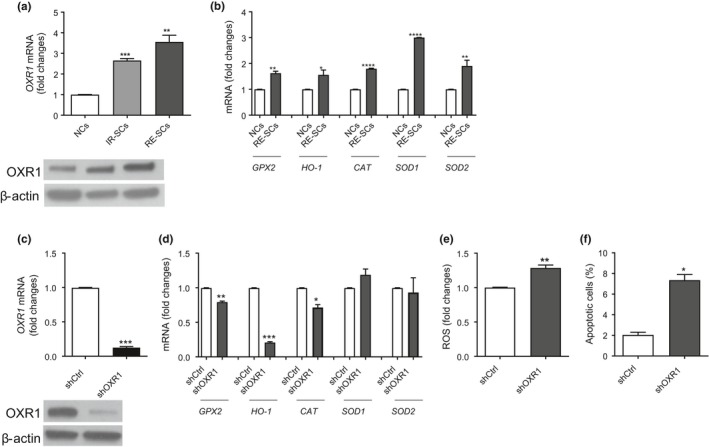
Knocking down OXR1 also induces apoptosis in RE‐SCs via downregulating the expression of antioxidant enzymes and increasing ROS production. (a) The levels of (top) *OXR1* mRNA and (bottom) OXR1 protein in NCs, IR‐SCs, and RE‐SCs. (b) Expression of *GPX2*,*HO‐1*,*CAT*,*SOD1*, and *SOD2* mRNA in NCs and RE‐SCs. Data in (a) and (b) are the means ± SE of fold changes in mRNA expression compared with NCs from two independent experiments. *p <* *0.05, **p <* *0.01, ***p <* *0.001, and ****p <* *0.0001 vs. NCs by unpaired *t* test. (c) The levels of (top) *OXR1* mRNA and (bottom) OXR1 protein in RE‐SCs transfected with shCtrl or shOXR1. (d) The levels of *GPX2*,*HO‐1*,*CAT*,*SOD1*, and *SOD2* mRNA in IR‐SCs transfected with shCtrl or shOXR1. Data in (c) and (d) are the means ± SE of fold changes in mRNA expression compared with shCtrl‐transfected cells from two independent experiments. *p <* *0.05, **p <* *0.01, and ***p <* *0.001 vs. shCtrl by unpaired *t* test. (e) The levels of intracellular ROS in RE‐SCs transfected with shCtrl or shOXR1. The data are presented as the mean ± SE of fold changes compared with shCtrl‐transfected RE‐SCs from two independent experiments. *p <* *0.05 vs. shCtrl by unpaired *t* test. (f) Percent apoptotic cells in RE‐SCs transfected with shCtrl or shOXR1 are presented as the mean ± SE of two independent experiments. *p <* *0.05 vs. shCtrl by unpaired *t* test

To determine whether OXR1 is a general senolytic target that is not specific to IR‐SCs, we examined the expression of *OXR1* in RE‐SCs generated after extensive replication of WI‐38 cells in vitro. We found that RE‐SCs, like IR‐SCs, also expressed increased levels of *OXR1* and its downstream targets (Figure 5a, b). Knocking down *OXR1* significantly reduced the expression of *GPX2, HO‐1,* and *CAT*, the known downstream targets of OXR1 (Figure 5c, d), and this increased ROS production and apoptosis in RE‐SCs (Figure 5e, f). These findings suggest that OXR1 is also important for RE‐SCs to counteract oxidative stress, which is in agreement with our observation that RE‐SCs are also sensitive to PL treatment (Figure 1b).

## 3 DISCUSSION

Resistance to apoptosis and oxidative stress is a hallmark of SCs (Childs, Baker, Kirkland, Campisi & Van Deursen, 2014; Childs et al., 2017) that can be exploited to develop senolytic agents that selectively kill SCs, assuming that the molecules that mediate the resistance can be identified. Indeed, we and others have recently discovered that the upregulation of the antiapoptotic Bcl‐2 family proteins is primarily responsible for the resistance of SCs to apoptosis, and Bcl‐2/xl/w inhibitors such as ABT‐263 are potent senolytic agents (Chang et al., 2016; Yosef et al., 2016; Zhu et al., 2016). Because long‐term treatment with a senolytic drug may be required to prevent/treat age‐related diseases and extend lifespan, there is a concern that Bcl‐2/xl/w inhibitors might be not safe for humans because of their known on‐target (thrombocytopenia) and off‐target toxicities (Roberts et al., 2011; Rudin et al., 2012; Vogler et al., 2011). Thus, it is important that we continue searching for novel senolytic targets to develop safer senolytic agents.

In the present study, we used an MS‐based proteomic approach to identify the direct protein targets of PL in SCs, hoping to find new senolytic targets that can be exploited for the development of a safer senolytic drug because PL is a relatively nontoxic, natural product (Adams et al., 2012; Raj et al., 2011; Wang et al., 2016). We designed a PL‐derived probe with senolytic properties similar to PL that can be tagged to resins via a bio‐orthogonal Click Reaction, allowing us to isolate proteins that interact covalently with the probe. Because it can be used with live cells, this approach allowed us to identify more biologically relevant targets than a traditional cell lysate‐based approach with a biotin‐labeled probe, which is not cell permeable and is not active in live cells as a senolytic agent (data not shown). With this approach, we identified 172 potential senolytic targets of PL. These targets have no overlap with known PL‐target proteins that were identified in cancer cell lysates with a biotin‐labeled PL probe (Raj et al., 2011), suggesting that PL may have different targets in SCs and cancer cells.

Among these possible senolytic targets of PL, we pursued OXR1 because it helps protect cells from oxidative stress by regulating several ROS‐detoxifying enzymes (Jaramillo‐Gutierrez et al., 2010; Oliver et al., 2011; Yang et al., 2015). Our studies showed that OXR1 is upregulated in both prematurely SCs induced by IR and replicative SCs in conjunction with the increased production of ROS and elevated expression of OXR1‐regulated ROS‐detoxifying enzymes such as GPX2, HO‐1, and CAT. In SCs, PL binds OXR1 directly and induces its degradation via the ubiquitin‐proteasome system; this results, in part, from a differential ability of SCs and NCs to take up PL. Genetic knockdown of *OXR1* resulted in the selective killing of SCs by disarming their defense against oxidative stress, because the induction of SC apoptosis by knocking down *OXR1* was associated with downregulation of *GPX2, HO‐1,* and *CAT* mRNA and increased production of ROS, and this was prevented by the antioxidant NAC. These findings and our observations that PL induces the proteasomal degradation of OXR1 and apoptosis of SCs suggest that OXR1 is a senolytic target that has the potential to be exploited for the development of novel senolytic agents, including PL analogs.

This study identifies OXR1 as a potential target of PL to mediate PL senolytic activity. However, PL can bind hundreds of proteins in SCs. It has yet to be determined whether other PL‐binding proteins also play a role in mediating the effect of PL on SCs. In addition, we observed that PL increased the levels of polyubiquitinated proteins in IR‐SCs (Figure 2g), suggesting that PL may also induce dysregulation of the ubiquitin‐proteasomal system and proteostasis in SCs. Whether the dysregulation induced by PL contributes to PL‐induced SC, apoptosis has yet to be investigated.

## 4 EXPERIMENTAL PROCEDURES

### 4.1 Cell culture and induction of senescence

Cell culture and senescence induction were performed as described before (Chang et al., 2016; Wang et al., 2016). See the details in Supporting Information Data S1.

### 4.2 Cell viability assays

Cell viability was measured by flow cytometry, as previously described (Chang et al., 2016; Wang et al., 2016). Dose–response curves were generated, and the half‐maximal effective concentrations (EC_50_ values) were calculated with GraphPad Prism 6 software.

### 4.3 Chemicals

PL was purchased from BioVision (catalog no. 1919‐10; Milpitas, CA).

### 4.4 Procedures for the synthesis of the PL probe, CTL probe, and PL‐biotin

See details in Supporting Information Data S1.

### 4.5 PL‐target protein pull down

The pull‐down experiments were carried out using the Click‐&‐Go™Protein Enrichment Kit (Product No. 1039, Bioconjugate Technology Company, Scottsdale, AZ) by following the protocol from the manufacturer. Briefly, nonsenescent WI38 cells (NCs), IR‐induced senescent WI38 cells (IR‐SCs), and replicative senescent WI38 cells (RE‐SCs) were plated on 10‐cm Petri dishes. The cells were incubated with PL probe or CTL probe at 5 μM for 5 hr, harvested, and lysed in a lysis buffer as described in the protocol. The concentration of the total protein was measured by NanoDrop. The same amount of total protein (4.48 mg in 800 μl) from each cell type was incubated with azide‐agarose resin in the presence of a copper catalyst solution overnight at room temperature, followed by reduction in 10 mM DTT (Sigma‐Aldrich) and alkylation in 40 mM iodoacetamide (Sigma‐Aldrich). The resin was washed, followed by on‐column protease digestion, and analyzed by LC‐MS/MS as described below. To exclude the nonspecific binding proteins, competitive‐binding experiments were performed in which IR‐SCs were incubated with 5 μM PL for 2 hr then incubated with 5 μM PL probe for 3 hr. In parallel, IR‐SCs were initially incubated with 5 μM PL probe for 2 hr then incubated with 5 μM PL for 3 hr. We repeated the CTL and PL probe binding assays and proteomics analyses two to three times for each cell type. PL selective and specific targets were identified using the combination of an enrichment ratio of ≥2 (i.e., the average spectra reads in IR‐SCs and RE‐SCs were ≥2‐fold higher than those in NCs) and a PL competition ratio of ≥2 (i.e., the spectra reads on cells treated with the PL probe followed by PL were ≥2‐fold higher than those in the cells treated with PL followed by the PL probe), as described in the manuscript.

### 4.6 LC‐MS/MS analysis and protein identification

LC‐MS/MS analysis and protein identification were performed after the procedure as described before (Sengupta et al., 2016). Briefly, trypsin was added to the solution to release peptides from the beads for affinity purification. Peptide products were then acidified in 0.1% Pierce formic acid (catalog no. 28905, Thermo Fisher Scientific, Grand Island, NY). The peptides were desalted using C18 Sep‐Pak and dried by speed‐vac. Tryptic peptides were separated by reverse‐phase Jupiter Proteo resin (Phenomenex, Torrance, CA) on a 200 × 0.075 mm column using a nanoAcquity UPLC system (Waters, Milford, MA). Peptides were eluted using a 30‐min gradient from 97:3 to 67:33 buffers A:B ratio [Buffer A = 0.1% formic acid, 0.5% acetonitrile; buffer B = 0.1% formic acid, 99.9% acetonitrile.]. Eluted peptides were ionized by electrospray (2.15 kV) followed by MS/MS analysis using higher‐energy collisional dissociation (HCD) on an Orbitrap Fusion Tribrid mass spectrometer (Thermo Scientific, San Jose, CA) in top‐speed data‐dependent mode. MS data were acquired using the FTMS analyzer in profile mode at a resolution of 240,000 over a range of 375 to 1,500 m/z. Following HCD activation, MS/MS data were acquired using the ion trap analyzer in centroid mode and normal mass range with precursor mass‐dependent normalized collision energy between 28.0 and 31.0. Proteins were identified by database search using Mascot (Matrix Science, Boston, MA) with a parent ion tolerance of 3 ppm and a fragment ion tolerance of 0.5 Da. Scaffold (Proteome Software, Portland, OR) was used to verify MS/MS‐based peptide and protein identifications. Peptide identifications were accepted if they could be established with less than 1.0% false discovery by the Scaffold Local false discovery rate (FDR) algorithm and contained at least two identified peptides. Protein probabilities were assigned by the Protein Prophet algorithm (Nesvizhskii, Keller, Kolker & Aebersold, 2003).

### 4.7 Raw data processing

All raw files were analyzed together using software MaxQuant (Cox & Mann, 2008) (version 1.5.3.30) as described before (Keilhauer, Hein & Mann, 2015) with minor modification. The derived peak list was searched with the built‐in Andromeda search engine (Cox et al., 2011) against the reference human proteome downloaded from Uniprot (http://www.uniprot.org/) on 04‐20‐2016 (176,494 sequences). Strict trypsin specificity was required with cleavage C‐terminal after K or R, allowing up to two missed cleavages. The minimum required peptide length was set to seven amino acids. Carbamidomethylation of cysteine was set as a fixed modification, and N‐acetylation of proteins N termini and oxidation of methionine were set as variable modifications. As no labeling was performed, multiplicity was set to 1. During the main search, parent masses were allowed an initial mass deviation of 4.5 ppm, and fragment ions were allowed a mass deviation of 0.5 Da. PSM and protein identifications were filtered using a target‐decoy approach at a FDR of 1%. The second peptide feature was enabled. The match between runs option was also enabled with a match time window of 0.7 min and an alignment time window of 20 min. Relative, label‐free quantification of proteins was performed using the MaxLFQ algorithm (Cox et al., 2014) integrated into MaxQuant. The parameters were as follows: Minimum ratio count was set to 1, the FastLFQ option was enabled, LFQ minimum number of neighbors was set to 3, and the LFQ average number of neighbors was set to 6, as per default. The “ProteinGroups” output file from MaxQuant is available in the supplement (Supporting Information Table S1).

### 4.8 Bioinformatics analysis

The GO enrichment and Kyoto Encyclopedia of Genes and Genomes (KEGG) pathway enrichment of identified PL‐target proteins were obtained from the DAVID Bioinformatics Resource (http://david.niaid.nih.gov). *p* Value <0.05 was used as a cutoff for the enriched items in both GO and KEGG pathway enrichment analyses.

### 4.9 Western blot analysis

Western blot analysis was performed according to our previous publication (Chang et al., 2016). See the details in Supporting Information Data S1.

### 4.10 PL‐OXR1 binding assays

#### 4.10.1 PL‐binding assay with cell lysate

A total of 1.2 mg of proteins from the lysates of IR‐SCs in 300 μl lysis buffer (Cat# BP‐115DG, Boston BioProducts, Ashland, MA) was incubated with the biotin‐labeled PL probe in the presence or absence of a fivefold excess of PL overnight at 4°C, and the mixture was then incubated with Pierce™ high capacity streptavidin agarose beads (20359, Thermo Scientific) for 30 min at room temperature. Unbound proteins were washed away, and the bound proteins were released by boiling in 1× SDS loading buffer at 95°C for 10 min. The released proteins were detected by Western blot analysis, as described above, with the OXR1 antibody (Cat# A30‐035A, Bethyl).

#### 4.10.2 PL‐binding assay with human OXR1 recombinant protein

Six hundred nanograms purified human recombinant OXR1 (NM_181354) protein (Cat# TP760961, OriGene, Rockville, MD) was incubated with biotin‐labeled PL probe in the absence or presence of a fivefold excess of unlabeled PL. The mixture was separated by SDS‐PAGE, and an immunoblot was performed with antibodies against biotin (Streptavidin (HRP), cat# Ab7403, Abcam) to detect OXR1 bound to the biotin‐labeled PL probe. An immunoblot of OXR1 from each reaction was included as an input control.

### 4.11 SA–β‐galactosidase (SA‐β‐gal) staining

See the procedure as described before (Chang et al., 2016; Wang et al., 2016). See the details in Supporting Information Data S1.

### 4.12 RNA extraction and real‐time PCR analysis

Cells were lysed, and total RNA was extracted using an RNeasy Mini Kit (catalog no. 74106, Qiagen, Germantown, MD). RNA (1 μg) was reverse‐transcribed with the Superscript II first strand synthesis kit (Invitrogen, Germantown, MD). Real‐time PCR was performed using specific primers (Supporting Information Table S5) and the SYBR Green ROX Mix (Thermo Scientific). The sequence of the primers is given below. All experiments were performed in two to three replicates, and data were normalized to the housekeeping gene *GAPDH*.

### 4.13 Co‐immunoprecipitation (Co‐IP) assay

IR‐SCs were treated with or without PL (10 μM) for 6 hr and then were harvested for Co‐IP assay using the Co‐IP kit (cat# 26149, Thermo Fisher Scientific) following the protocol from the manufacturer. MG132 (1 μM) was added during treatment to inhibit protein degradation. Briefly, 5 μg rabbit anti‐OXR1 antibody (Cat# A302‐036A, Bethyl) or rabbit IgG control antibody (Cat# 2729S, CST) was immobilized to the column, and 750 μg total lysate protein was added to the resin in the column. The precipitated protein was analyzed for OXR1 ubiquitination by Western blot analysis using anti‐ubiquitin antibody (Cat# 3936S, CST) or anti‐OXR1 antibody (Cat# A302‐035A, Bethyl). Cell lysates were also immunoblotted with antibodies to OXR1 or β‐actin as indicated.

### 4.14 Analysis of intracellular levels of PL by Click Chemistry

NCs and IR‐SCs were incubated with 5 μM PL probe or vehicle for 5 hr, and Alexa Fluor azide 488 was added to detect the PL uptake efficiency following the Click‐It EdU imaging kit protocol (C10337, Thermo Fisher Scientific). The levels of PL in cells were visualized by microscopy then quantified by flow cytometry and ImageStream flow cytometry. For microscopy, images were taken with the ZEISS image system as described before (Chang et al., 2016). For flow cytometry, after fixing and staining, Alexa Fluor 488 conjugated to the PL probe inside the cells was measured with a BD LSR II flow cytometer (BD Biosciences, San Jose, CA). The MFI of Alexa Fluor 488‐conjugated PL probe was normalized by cell size which was measured with FSC measurement. For ImageStream flow cytometry, Alexa Fluor 488 conjugated to the PL probe was quantified with ImageStreamX Mark II System (Amnis, Merck Millipore, Germany) following the instructions from the manufacturer, and then normalized by cell size which was measured by the average area of cells.

### 4.15 Knockdown of *OXR1* with short hairpin RNAs (shRNAs)

We followed the procedure as described before (Chang et al., 2016) with some modifications. See the details in Supporting Information Data S1.

### 4.16 ROS measurement and apoptosis assay

ROS and apoptosis were measured with flow cytometry as previously described (Wang et al., 2016). See the details in Supporting Information Data S1.

## CONFLICT OF INTEREST

GZ, XL, YW, JC, XZ, and DZ filed a patent application for the use of PL and PL analogs as anti‐aging agents. In addition, JC and DZ are cofounders of and GZ, JC, and DZ are advisors to Unity Biotechnology, which develops small molecule, senolytic drugs. RML is employed by Unity Biotechnology.

## AUTHORS’ CONTRIBUTION

X(in)Z and SZ performed the majority of the experiments. GZ designed the PL and CTL probe. XL and X(uan)Z synthesized the probes. YW and JC assisted with some of the experiments. SGM and AJT performed the MS proteomics analysis. YH and DL performed gene ontology and pathway analyses. X(in)Z, SZ, RML, JC, JW, GZ, and DZ contributed to experimental design, data analysis, and manuscript preparation.
