# Supplementary material for: Oxidation resistance 1 is a novel senolytic target
Source: Aging Cell. 2018 May 15;17(4):e12780. doi: 10.1111/acel.12780 (PMC6052462; doi:10.1111/acel.12780)
Supplement: Supplementary file 1 [file ACEL-17-na-s001.doc]

**Fig. S1**

**Fig. S1.** **Synthetic scheme for the PL probe, CTL probe, and PL-biotin.** *Reagents and conditions*: a) AlCl_3_, CH_2_Cl_2_, 0°C; b) PPh_3_, DIAD, but-3-yn-1-ol, THF, rt; c) pivaloyl chloride, TEA, THF, 0°C; d) *n*-BuLi, 5,6-dihydropyridin-2(1*H*)-one, THF, -78°C; e) 1-hydroxypyrrolidine-2,5-dione, EDC, DMF, rt; f) PPh_3_, DIAD, 3-azidopropan-1-ol, THF, rt; g) (Boc)_2_O, DIPEA, CH_2_Cl_2_, rt; h) hex-5-ynoic acid, EDC, HOBt, DMF, rt; i) 2 M HCl in Et_2_O, rt; j) TEA, DMF, rt; k) CuSO_4_, sodium ascorbic acid, *t*-BuOH/H_2_O, rt
