# Supplementary material for: Oxidation resistance 1 is a novel senolytic target
Source: Aging Cell. 2018 May 15;17(4):e12780. doi: 10.1111/acel.12780 (PMC6052462; doi:10.1111/acel.12780)
Supplement: Supplementary file 2 [file ACEL-17-na-s002.doc]

**Fig. S2**

**Fig. S2.** **ROS production and sensitivity to H_2_O_2_ in NCs and SCs.** (*A*) Compared to NCs, IR-SCs exhibit increases in autofluorescence (without DHR) and produce increased levels of ROS detected by DHR. Representative histograms of flow cytometric analyses for (left) NCs and (middle) IR-SCs; (right) fold changes in ROS production were calculated as ratio of MFI in NCs after subtracting autofluorescence. Data in the bar graph are the mean ± SE (*n* = 3). **p* < 0.05 vs. NCs by unpaired *t*-test. (*B*) IR-SCs are more resistant to H_2_O_2_ cytotoxicity than NCs according to the half-maximal effective concentrations (EC_50_ values) of H_2_O_2_.
