# Supplementary material for: Oxidation resistance 1 is a novel senolytic target
Source: Aging Cell. 2018 May 15;17(4):e12780. doi: 10.1111/acel.12780 (PMC6052462; doi:10.1111/acel.12780)
Supplement: Supplementary file 7 [file ACEL-17-na-s007.doc]

**Table S5** Sequences of the primers used for qRT–PCR

| **Genes** | **Forward sequences** | **Reverse sequences** |
| --- | --- | --- |
| *OXR1* | ACATTCTGTCCGGAGTTTGAG | CACCACCGAAAGCTAGTGAA |
| *SOD1* | TTGGGCAATGTGACTGCTGACAAA | GGGCGATCCCAATTACACCACAA |
| *SOD2* | CTGATTTGGACAAGCAGCAA | CTGGACAAACCTCAGCCCTA |
| *GPX2* | AGAATGATGGCACCTTCCTAAA | TCCACACCTGCCCTTTATTG |
| *HO-1* | TCTTGGCTGGCTTCCTTAC | CATAGGCTCCTTCCTCCTTTC |
| *Catalase* | CTGGAGCACAGCATCCAATA | TCATTCAGCACGTTCACATAGA |
| *GAPDH* | GACCACTTTGTCAAGCTCATTTC | CTCTCTTCCTCTTGTGCTCTTG |
