# Supplementary material for: Oxidation resistance 1 is a novel senolytic target
Source: Aging Cell. 2018 May 15;17(4):e12780. doi: 10.1111/acel.12780 (PMC6052462; doi:10.1111/acel.12780)
Supplement: Supplementary file 8 [file ACEL-17-na-s008.doc]

**Supporting Information**

**Oxidation resistant 1 is a novel senolytic target**

Xin Zhang, Suping Zhang, Xingui Liu, Yingying Wang, Jianhui Chang, Xuan Zhang, Samuel G. Mackintosh, Alan J. Tackett, Yonghan He, Dongwen Lv, Remi-Martin Laberge, Judith Campisi, Jianrong Wang, Guangrong Zheng, Daohong Zhou

**SI Materials and Methods**

**Cell culture and induction of senescence.** Briefly, human WI38 fibroblasts (WI38, catalog no. CCL-75, American Type Culture Collection, Manassas, VA) were cultured in a complete Dulbecco’s Modified Eagle Medium (DMEM, catalog no. 12430, Gibco, Waltham, MA) supplemented with 10% fetal bovine serum (catalog no. 16000044, Atlanta Biologicals, Norcross, GA), 100 U/ml penicillin, and 100 µg/ml streptomycin (catalog no. P4333, Sigma-Aldrich, St. Louis, MO) in a humidified incubator at 37°C with 5% CO_2_. Low-passage WI38 (< 25 passages) cells were used as controls or for the induction of senescence. To induce replicative senescence, WI38 cells were subcultured until they stopped dividing and became senescent (after approximately 38 passages). To induce senescence with IR, WI38 cells, at roughly 70% confluency, were exposed to 15 Gy of IR in a J. L. Shepherd Model Mark I ^137^Cesium γ-irradiator (J. L. Shepherd, Glendale, CA) at a dose rate of 1.080 Gy/min. Three days after irradiation, cells were passaged once at a 1:3 dilution. WI38 cells became fully senescent 10 days after irradiation.

**Procedures for the Synthesis of the PL probe, CTL probe, and PL-biotin.**

***General Methods.*** Dry solvents (THF, CH_2_Cl_2_, and DMF) were obtained from a solvent purification system. All other reagents and solvents obtained from commercial sources were used without further purification. If dry and air-free conditions were required, reactions were performed in oven-dried glassware (130ºC) under a positive pressure of argon. Flash chromatography was performed using silica gel (230-400 mesh) as the stationary phase. Reaction progress was monitored by thin layer chromatography (silica-coated glass plates) and visualized under UV light and after exposure to iodine vapor; it was also assayed by GC-MS, LC-MS, or TLC-MS. NMR spectra were recorded in CDCl_3_ or CD_3_OD at 400 MHz for ^1^H and 100 MHz for ^13^C NMR. Chemical shifts δ are given in ppm using tetramethylsilane as an internal standard. Multiplicities of ^1^H NMR signals are designated as singlet (s), broad singlet (*br* s), doublet (d), doublet of doublets (dd), triplet (t), quartet (q), and multiplet (m). ^13^C NMR spectra were determined with broadband decoupling.

***Preparation of 1-[(2E)-3-(4-(but-3-yn-1-yloxy)-3,5-dimethoxyphenyl)prop-2-enoyl]-5,6-dihydropyridin-2(1H)-one (PL probe).*** 1-[(2*E*)-3-(4-hydroxy-3,5-dimethoxyphenyl)prop-2-enoyl]-5,6-dihydropyridin-2(1*H*)-one (compound **1**) was synthesized as reported (3, 4). To a solution of compound **1** (60 mg, 0.2 mmol) in THF (3 mL) was added but-3-yn-1-ol (15 mg, 0.2 mmol), triphenylphosphine (PPh_3_) (78 mg, 0.3 mmol), and diisopropyl azodicarboxylate (DIAD) (60 mg, 0.3 mmol). The resulting mixture was allowed to stir at room temperature overnight. The solvent was evaporated under vacuum, and the resulting residue was purified by silica gel flash column chromatography (eluted with ethyl acetate/hexanes 1:1) to afford PL probe (40 mg, yield 57%) as a white solid. ^1^H NMR (400 MHz, CDCl_3_) δ 7.65 (d, *J* = 15.6 Hz, 1H), 7.41 (d, *J* = 15.6 Hz, 1H), 6.88–6.98 (m, 1H), 6.78 (s, 2H), 5.97–6.08 (m, 1H), 4.12 (t, *J* = 7.5 Hz, 2H), 4.02 (t, *J* = 6.5 Hz, 2H), 3.86 (s, 6H), 2.64 (td, *J* = 7.5, 2.7 Hz, 2H), 2.38–2.52 (m, 2H), 1.97 (t, *J* = 2.7 Hz, 1H); ^13^C NMR (100 MHz, CDCl_3_) δ 168.92, 165.93, 153.56, 145.65, 143.76, 138.44, 131.05, 125.89, 121.30, 105.50, 80.76, 71.08, 69.63, 56.25, 41.74, 24.90, 20.14; MS (ES^+^) *m/z* 356.4 [M+H^+^], 378.3 [M+Na^+^].

***Preparation of 1-(3-(3,4,5-trimethoxyphenyl)propanoyl)-5,6-dihydropyridin-2(1H)-one (compound 3).*** To a solution of 3-(3,4,5-trimethoxyphenyl)propanoic acid (compound **2**) (2.23 g, 9.3 mmol) in dry THF (10 mL) was added triethylamine (1.68 mL, 12.1 mmol) and trimethyl acetic chloride (1.25 mL, 11.2 mmol) at 0°C. The resulting mixture was stirred at the same temperature for 45 min then filtered under a nitrogen atmosphere. In a separate flask, 5,6-dihydropyridin-2(1*H*)-one (300 mg, 3.1 mmol) was dissolved in dry THF (10 mL) and cooled to -78 °C. Then, *n*-BuLi (1.6 M in hexane, 3.36 mmol) was added dropwise. After stirring for 45 min, one-third of the above filtrate was added dropwise. After stirring for another 45 min at -78°C, the mixture was quenched with saturated aqueous ammonium chloride and extracted with ethyl acetate (× 3). The combined organic layers were washed with brine, dried over sodium sulfate, filtered, and concentrated under vacuum. The resulting crude residue was purified by silica gel flash column chromatography (eluted with ethyl acetate/hexanes 1:1) to afford compound **3** (510 mg, yield 51%) as a white solid. ^1^H NMR (400 MHz, CDCl_3_) δ 6.77–6.98 (m, 1H), 6.47 (s, 2H), 5.99 (d, *J* = 9.7 Hz, 1H), 3.97 (t, *J* = 6.5 Hz, 2H), 3.85 (s, 6H), 3.81 (s, 3H), 3.25 (t, *J* = 7.7 Hz, 2H), 2.94 (t, *J* = 7.7 Hz, 2H), 2.34–2.45 (m, 2H); ^13^C NMR (100 MHz, CDCl_3_) δ 175.66, 165.52, 153.21, 145.40, 137.08, 136.42, 126.00, 105.54, 60.98, 56.19, 41.16, 41.06, 31.70, 24.78.

***Preparation of 1-(3-(4-hydroxy-3,5-dimethoxyphenyl)propanoyl)-5,6-dihydropyridin-2(1H)-one (compound 4).*** To a solution of 1-(3-(3,4,5-trimethoxyphenyl)propanoyl)-5,6-dihydropyridin-2(1*H*)-one (compound **3**) (200 mg, 0.63 mmol) in methylene chloride (5 mL) was added AlCl_3_ (600 mg, 4.5 mmol) at 0°C. The resulting mixture was stirred at room temperature overnight, quenched with water at 0°C, and extracted with ethyl acetate (× 3). The combined organic layers were washed with brine, dried over sodium sulfate, filtered, and concentrated under vacuum. The resulting crude residue was purified by silica gel flash column chromatography (eluted with ethyl acetate/hexanes 1:1) to afford compound **4** (60 mg, yield 31%) as a white solid. ^1^H NMR (400 MHz, CDCl_3_) δ 6.85–6.93 (m, 1H), 6.48 (s, 2H), 5.99 (d, *J* = 9.7 Hz, 1H), 5.43 (*br* s, 1H), 3.97 (t, *J* = 6.5 Hz, 2H), 3.87 (s, 6H), 3.24 (t, *J* = 7.7 Hz, 2H), 2.92 (t, *J* = 7.7 Hz, 2H), 2.35–2.45 (m, 2H); ^13^C NMR (100 MHz, CDCl_3_) δ 175.68, 165.48, 146.97, 145.37, 133.02, 132.34, 125.92, 105.23, 56.35, 41.26, 41.11, 31.46, 24.73.

***Preparation of 1-(3-(4-(but-3-yn-1-yloxy)-3,5-dimethoxyphenyl)propanoyl)-5,6-dihydropyridin-2(1H)-one (CTL probe).*** To a solution of 1-(3-(4-hydroxy-3,5-dimethoxyphenyl)propanoyl)-5,6-dihydropyridin-2(1H)-one (compound **4**) (70 mg, 0.23 mmol) in dry THF (3 mL) was added but-3-yn-1-ol (16 mg, 0.23 mmol), PPh_3_ (90.2 mg, 0.35 mmol), and DIAD (70 mg, 0.35 mmol). The resulting mixture was allowed to stir at room temperature overnight. Solvent was removed under vacuum, and the resulting crude residue was purified by silica gel flash column chromatography (eluted with ethyl acetate/hexanes 1:1) to afford the CTL probe (25 mg, yield 30%) as a white solid. ^1^H NMR (400 MHz, CDCl_3_) δ 6.86–6.95 (m, 1H), 6.46 (s, 2H), 5.99 (dt, *J* = 9.7, 1.8 Hz, 1H), 4.07 (t, *J* = 7.6 Hz, 2H), 3.97 (t, *J* = 6.5 Hz, 2H), 3.83 (s, 6H), 3.20–3.29 (m, 2H), 2.93 (t, *J* = 7.7 Hz, 2H), 2.65 (td, *J* = 7.6, 2.7 Hz, 2H), 2.35–2.43 (m, 2H), 1.97 (s, 1H); ^13^C NMR (100 MHz, CDCl_3_) δ 175.63, 165.50, 153.42, 153.29, 145.40, 137.37, 134.69, 125.97, 105.54, 81.03, 71.01, 69.46, 56.18, 41.15, 41.01, 31.69, 24.77, 20.04. MS (ES^+^) *m/z* 358.4 [M+H^+^], 380.3 [M+Na^+^].

***Preparation of biotin succinimide (compound 5).*** To a solution of *D*-biotin (500 mg, 2.05 mmol) in anhydrous DMF (20 mL) was added *N*-hydroxysuccinimide (258 mg, 2.25 mmol) and 1-ethyl-3-(3-dimethylaminopropyl) carbodiimide hydrogen chloride (EDC∙HCl) (471 mg, 2.46 mmol). The resulting suspension was stirred for 40 h at room temperature, filtered, washed with methanol, and concentrated under vacuum to afford compound **5** (341 mg, yield 49%) as a white solid. MS (ES^+^) *m/z* 342.2 [M+H^+^], 364.0 [M+Na^+^].

***Preparation of 1-[(2E)-3-(4-(3-azidopropoxy)-3,5-dimethoxyphenyl)prop-2-enoyl)-5,6-dihydropyridin-2(1H)-one (compound 6).*** To a solution of compound **1** (100 mg, 0.33 mmol) in THF (3 mL) at 0°C was added 3-azidopropan-1-ol (33.3 mg, 0.33 mmol), PPh_3_ (130 mg, 0.5 mmol), and DIAD (99.6 mg, 0.5 mmol). The resulting mixture was allowed to stir at room temperature overnight. Solvent was removed under vacuum, and the resulting crude residue was purified by silica gel flash column chromatography (eluted with ethyl acetate/hexanes 1:1) to afford compound **6** (95 mg, yield 75%) as a white solid. ^1^H NMR (400 MHz, CDCl_3_) δ 7.67 (d, *J* = 15.5 Hz, 1H), 7.42 (d, *J* = 15.5 Hz, 1H), 6.87–7.01 (m, 1H), 6.79 (s, 2H), 6.04 (d, *J* = 9.7 Hz, 1H), 4.05 (dt, *J* = 13.0, 6.2 Hz, 4H), 3.87 (s, 6H), 3.60 (t, *J* = 6.7 Hz, 2H), 2.47 (dd, *J* = 10.7, 4.7 Hz, 2H), 1.93–2.02 (m, 2H); ^13^C NMR (100 MHz, CDCl_3_) δ 168.97, 165.96, 153.59, 145.65, 143.86, 138.84, 130.91, 125.92, 121.24, 105.49, 70.13, 56.22, 48.51, 41.76, 29.82, 24.92.

***Preparation of tert-butyl (2-(2-(2-(hex-5-ynamido)ethoxy)ethoxy)ethyl)carbamate (compound 9).*** tert-Butyl (2-(2-(2-aminoethoxy)ethoxy)ethyl)carbamate (compound **8**) was synthesized from 2,2'-(ethane-1,2-diylbis(oxy))bis(ethan-1-amine) (compound **7**) as reported (5). To a solution of **7** (496 mg, 2 mmol) in DMF (6 mL) was added hex-5-ynoic acid (246 mg, 2.2 mmol), hydroxybenzotriazole (HOBt) (405 mg, 3 mmol), EDC∙HCl (575 mg, 3 mmol), and DIPEA (0.87 mL, 5 mmol). The resulting mixture was stirred at room temperature overnight then concentrated under vacuum. The resulting residue was diluted with ethyl acetate and washed with 1M aqueous HCl. The organic layer was collected, dried over sodium sulfate, filtered, and concentrated under vacuum. The resulting crude residue was purified by silica gel flash column chromatography (eluted with ethyl acetate/methanol/NH_3_∙H_2_O 5/1/0.1) to afford compound **9** (670 mg, yield 98%). ^1^H NMR (400 MHz, CDCl_3_) δ 6.15 (*br* s, 1H), 5.00 (*br* s, 1H), 3.60 (s, 4H), 3.54 (t, *J* = 5.0 Hz, 4H), 3.42–3.48 (m, 2H), 3.23–3.36 (m, 2H), 2.33 (t, *J* = 7.4 Hz, 2H), 2.23 (t, *J* = 5.5 Hz, 2H), 1.96 (s, 1H), 1.80–1.90 (m, 2H), 1.43 (s, 9H).

***Preparation of N-(2-(2-(2-(5-((3aS,4S,6aR)-2-oxohexahydro-1H-thieno[3,4-d]imidazol-4-yl) pentanamido)ethoxy)ethoxy)ethyl)-hex-5-ynamide (11).*** To a solution of compound **9** (200 mg, 0.58 mmol) in ethyl acetate (2 mL) was added HCl (2M in diethyl ether, 1.2 mL, 2.4 mmol). The resulting mixture was stirred at room temperature, and the solvent was removed under vacuum to afford *N*-(2-(2-(2-aminoethoxy)ethoxy)ethyl)hex-5-ynamide (compound **10**) as a white solid which was used directly for the next step. To a solution of **10** in DMF (5 mL) was added biotin succinimide (compound **5**) (179 mg, 0.52 mmol) and triethylamine (0.25 mL, 1.75 mmol). The resulting mixture was stirred at room temperature overnight and then concentrated under vacuum. The resulting crude residue was purified by silica gel flash column chromatography (eluted with ethyl acetate/methanol/NH_3_∙H_2_O 5/1/0.1) to afford compound **11** (160 mg, yield 59%). ^1^H NMR (400 MHz, CD_3_OD) δ 8.05 (*br* s, 2H), 4.52 (dd, *J* = 7.7, 5.0 Hz, 1H), 4.33 (dd, *J* = 7.8, 4.4 Hz, 1H), 3.64 (s, 4H), 3.57 (t, *J* = 5.5 Hz, 4H), 3.39 (q, *J* = 5.5 Hz, 4H), 3.19–3.27 (m, 1H), 2.95 (dd, *J* = 12.8, 5.0 Hz, 1H), 2.73 (d, *J* = 12.8 Hz, 1H), 2.34 (t, *J* = 7.5 Hz, 2H), 2.20–2.30 (m, 5H), 1.55–1.89 (m, 6H), 1.47 (dd, *J* = 15.0, 7.5 Hz, 2H); ^13^C NMR (100 MHz, CD_3_OD) δ 176.15, 175.49, 166.03, 84.19, 71.26, 70.61, 70.57, 70.26, 63.34, 61.61, 56.98, 41.05, 40.25, 36.73, 35.80, 29.74, 29.49, 26.84, 25.94, 18.63.

***Preparation of N-(2-(2-(2-(4-(1-(3-(2,6-dimethoxy-4-((E)-3-oxo-3-(6-oxo-3,6-dihydropyridin-1(2H)-yl)prop-1-en-1-yl)phenoxy)propyl)-1H-1,2,3-triazol-4-yl)butanamido)ethoxy)ethoxy)ethyl)-5-((3aS,4S,6aR)-2-oxohexahydro-1H-thieno[3,4-d]imidazol-4-yl)pentanamide (PL-biotin).*** To a solution of compound **11** (40 mg, 0.085 mmol) in *t*-BuOH (2 mL) was added 1-[(2*E*)-3-(4-(3-azidopropoxy)-3,5-dimethoxyphenyl)prop-2-enoyl)-5,6-dihydropyridin-2(1H)-one (compound **6**) (35 mg, 0.091 mmol) and CuSO_4_∙5H_2_O (4.5 mg, 0.0018 mmol), followed by the addition of aqueous sodium ascorbate (9 mg in 2 mL H_2_O, 0.0045 mmol). The resulting mixture was stirred at room temperature under a nitrogen atmosphere overnight. Water was added, and the mixture was extracted with ethyl acetate (× 3). The combined organic layers were washed with brine, dried over sodium sulfate, filtered, and concentrated. The resulting crude residue was purified by silica gel flash column chromatography (eluted with methylene chloride/methanol 10/1) to afford PL-biotin (50 mg, yield 68%) as a white solid. ^1^H NMR (400 MHz, CD_3_OD) δ 7.83 (s, 1H), 7.62 (d, *J* = 15.6 Hz, 1H), 7.39 (d, *J* = 15.6 Hz, 1H), 7.04–7.13 (m, 1H), 6.93 (s, 2H), 6.02 (dt, *J* = 9.7, 1.7 Hz, 1H), 4.67 (t, *J* = 6.9 Hz, 2H), 4.49 (dd, *J* = 7.8, 4.8 Hz, 1H), 4.30 (dd, *J* = 7.8, 4.5 Hz, 1H), 3.95–4.04 (m, 4H), 3.89 (s, 6H), 3.62 (s, 4H), 3.55 (q, *J* = 5.6 Hz, 4H), 3.36 (q, *J* = 5.7 Hz, 4H), 3.16–3.23 (m, 1H), 2.92 (dd, *J* = 12.7, 5.0 Hz, 1H), 2.68–2.78 (m, 3H), 2.52 (qd, *J* = 6.2, 1.7 Hz, 2H), 2.14–2.33 (m, 6H), 1.97–2.02 (m, 2H), 1.53–1.81 (m, 4H), 1.44 (dd, *J* = 15.2, 7.4 Hz, 2H); ^13^C NMR (100 MHz, CD_3_OD) δ 176.10, 175.60, 170.52, 167.72, 166.06, 154.90, 148.42, 146.36, 144.12, 139.77, 132.45, 125.86, 123.90, 122.74, 106.54, 71.27, 70.60, 70.59, 63.34, 61.59, 57.02, 56.67, 43.04, 41.09, 40.30, 40.26, 36.74, 36.23, 31.82, 29.76, 29.48, 26.83, 26.76, 25.74; MS (ES^+^) *m/z* 876.8 [M+Na^+^].

**Western blot analysis.** Briefly, cells were lysed in RIPA buffer with EDTA and EGTA (catalog no. BP-115DG, Boston BioProducts, Ashland, MA), supplemented with 1% Phosphatase Inhibitor Cocktail 3 (catalog no. P0044, Sigma-Aldrich) and 1% Protease Inhibitor Cocktail (catalog no. P8340, Sigma-Aldrich). An equal amount of protein (30 µg/lane) from each cell extract was resolved on a 4–20% Mini-PROTEAN® TGX™ Precast Protein Gels (Bio-Rad, Hercules, CA). Proteins were transferred to a NOVEX PVDF membrane (catalog no. LC2002, Thermo Scientific). The membranes were blocked with 5% nonfat milk in TBST blocking buffer and probed with primary antibodies to OXR1 (catalog no. A302-035A, Bethyl Laboratories Inc, Montgomery, TX), β-actin (catalog no. SC-1615, Santa Cruz Biotechnology, Dallas, TX), and ubiquitin (catalog no. 646302, BioLegend, San Diego, CA) at a predetermined optimal concentration overnight at 4°C. The secondary antibodies (anti-rabbit or anti-goat) (Jackson ImmunoResearch Europe, Suffolk, UK) were conjugated to horseradish peroxidase. Signals were detected with the ECL system (catalog no. WBKLS0100, EMD Millipore, Newmarket, Suffolk, UK) and recorded with autoradiography (Pierce Biotech, Rockford, IL).

**Knockdown of *OXR1* with short hairpin RNAs (****shRNAs).** Briefly, control lentiviral pLKO.1 vectors and pLKO.1 vectors containing shRNAs specific for human *OXR1* (TRCN0000159799, TRCN0000160280) were obtained from Sigma-Aldrich. Lentivirus was produced after transient infection of human embryonic kidney (HEK) 293T cells with individual lentiviral vectors along with the packaging plasmids pCMV-VSV-G and psPAX2 (Addgene, Cambridge, MA) using FuGEN6 (E2691, Promega, Madison, WI) as the infection reagent following the Promega protocol. The supernatants containing viral particles were collected 48 h and 72 h after the infection and filtered through a 0.22 μm filter. The viral particles were concentrated by ultra-centrifugation at 26,000 rpm at 4ºC for 100 mins. The concentrated virus was resuspended in culture medium. The virus titer was determined with the QuickTiter Lentivirus Quantitation Kit (VPK-112, Cell Biolabs, Inc., San Diego, CA). To establish *OXR1*-knockdown cells, NCs, IR-SCs, or RE-SCs were plated in 10 cm petri-dishes and incubated with the virus in 5 ml serum-free medium for 8 hours followed by changing into complete medium. At 48 hours post-transduction, stably transduced cells were selected with puromycin (2 µg/ml) for 3 days. OXR1 knockdown was confirmed by real-time PCR and western blot before the cells were used in an experiment.

**ROS measurement.**  In brief, cells were trypsinized and washed with PBS and then resuspended in PBS containing 1 µM dihydrorhodamine 123 (DHR 123, catalog no. D632, Thermo Fisher Scientific) and incubated at room temperature for 30 min. After incubation, the cells were washed with PBS and assayed by flow cytometry. Mean fluorescence intensity (MFI) was determined with a BD LSR II flow cytometer.

**Apoptosis assay.** In brief, after *OXR1* knockdown, apoptosis was detected in NCs or SCs in the presence or absence of NAC/H_2_O_2_ treatment (NAC, 2 mM; catalog no. 138061, Sigma-Aldrich). At the desired time, cells were harvested and washed twice with Annexin V binding buffer and then stained with Alexa Fluor 647-Annexin V (1: 50, catalog no. 640912, BioLegend, San Diego, CA) and propidium iodide (PI, 10 µg/ml, catalog no. P4170, Sigma-Aldrich) for 30 min. Then, the stained cells were analyzed with a BD LSR II flow cytometer.
